# Learning Lessons from Adverse Drug Reactions in Children

**DOI:** 10.3390/children3010001

**Published:** 2016-01-08

**Authors:** Helen M. Sammons, Imti Choonara

**Affiliations:** University of Nottingham, Division of Medical Sciences and Graduate Entry Medicine, Derbyshire Children’s Hospital, Derby DE22 3DT, UK

**Keywords:** drug toxicity, children, drug metabolism, rational drug use

## Abstract

Drug toxicity is, unfortunately, a significant problem in children both in the hospital and in the community. Drug toxicity in children is different to that seen in adults. At least one in 500 children will experience an adverse drug reaction each year. For children in hospital, the risk is far greater (one in ten). Additionally, different and sometimes unique adverse drug reactions are seen in the paediatric age groups. Some of the major cases of drug toxicity historically have occurred in neonates. It is important that we understand the mechanism of action of adverse drug reactions. Greater understanding alongside rational prescribing should hopefully reduce drug toxicity in children in the future.

## 1. Introduction

Drug toxicity is a common clinical problem in paediatric patients of all ages. An adverse drug reaction (ADR) is defined by the World Health Organization (WHO) as “a response to a medicine which is noxious and unintended and which occurs at doses normally used in man” [1]. At least one in 500 children will experience an ADR each year [2]. One in ten children in hospital will experience an ADR [3]. It is impossible to prevent all ADRs, however there is a need to try and minimise drug toxicity. This can be achieved by using medicines rationally and prescribing medicines only when there is an evidence base that suggests they are effective. Additionally, one can learn lessons from some of the major ADRs that have occurred in paediatric patients. By understanding the mechanisms of actions of drug toxicity, one can then hopefully recognise potential drug toxicity associated with certain medicines. The aim of this paper is to review the mechanisms of drug toxicity using historical examples of adverse drug reactions in children. Such information will help health professionals use medicines more safely in the future.

## 2. Percutaneous Drug Toxicity

There have been several ADRs that have occurred in neonates and young infants due to percutaneous absorption of a variety of compounds [4]. Neonates and young infants have a higher surface area to weight ratio than children so medicines that are administered topically result in greater relative exposure. Transcutaneous diffusion of small molecules is also facilitated in this age group by a thinner *stratum corneum* and a greater water content in the dermis. In 1886 a letter was published in the British Medical Journal highlighting the toxicity of aniline dye in newborn infants in a workhouse [5]. Seventeen babies developed cyanosis due to methaemoglobinemia following cutaneous exposure to the aniline dye which was used to stamp the name of the institution (Marylebone Workhouse, UK) on their nappies. Percutaneous toxicity is not restricted to neonates. It has also been reported in infants and children. Thirty six infants and young children died in France in the early eighties following contamination of a talc baby powder with hexachlorophene [6]. Other examples of percutaneous toxicity include toxicity with iodine [7], alcohol [8], and nicotine [9] (Table 1).

**Table 1:** Mechanisms of adverse drug reactions in paediatric patients.

Mechanism	Drug	ADR
Percutaneous absorption	Aniline dye	Methaemoglobinaemia
	Hexachlorophene	Neurotoxicity
	Iodine	Hypothyroidism
	Alcohol	Metabolic acidosis
	Nicotine	Nausea/vomiting
Bilirubin displaced from albumin	Sulfisoxazole	Kernicterus
Impaired metabolism	Chloramphenicol	Grey baby syndrome
Abnormal metabolism	Valproic acid	Hepatotoxicity
Excipient toxicity	Benzyl alcohol	Multi-organ failure
	Diethylene glycol	
Drug interaction	Ceftriaxone and calcium containing solutions	Calcium precipitation
	Lamotrigine and valproic acid	Skin reactions
Polypharmacy	Valproic acid	Hepatotoxicity
Unknown	Salicylates	Reye’s syndrome
	Propofol	Metabolic acidosis and lipaemia
	Vigabatrin	Visual field defects
	Corticosteroids	Growth suppression Neurotoxicity
	Macrolides	Pyloric stenosis

ADR: adverse drug reaction.

- Percutaneous drug toxicity is more likely to be a significant problem in neonates and infants, than other age groups.

## 3. Protein Binding

In 1956 a clinical trial in the USA reporting two different antibiotic regimes in sick neonates described a higher mortality rate associated with an increase in kernicterus among one of the groups [10]. The authors were unable to explain their findings but fortunately reported the increased mortality associated with the use of a combination of penicillin and sulfisoxazole (a sulphonamide). The other group received oxytetracycline. The infants who received the combination of penicillin and sulfisoxazole were at greater risk of kernicterus and the associated seizures, opisthotonus, spasticity, and poor feeding.

Subsequent studies several years later demonstrated that sulphonamides are highly protein bound [11]. They, therefore, displace bilirubin from albumin causing an increase in the free fraction of bilirubin in the plasma. It is the free fraction of bilirubin (unconjugated) that is responsible for the development of kernicterus. Protein binding of medicines is not usually a clinical problem but in sick preterm infants with high bilirubin levels it is clearly a significant issue. It is important, therefore, to avoid highly protein bound medicines in neonates.

- Avoid highly protein bound medicines in sick neonates (eg.sulphonamides, ceftriaxone).

## 4. Ontogeny of Drug Metabolism

In 1959, the death of three newborn infants in Cincinnati, USA was reported following the administration of chloramphenicol [12]. The babies developed abdominal distension, vomiting, cyanosis, and cardiovascular collapse (grey baby syndrome). A year later it was demonstrated that the metabolism of chloramphenicol is impaired in neonates [13]. This impaired drug metabolism illustrates the importance of reducing the dose of chloramphenicol in neonates. At the time that chloramphenicol was initially used in neonates there had been no studies of drug metabolism in the newborn infant. Subsequent studies have shown that many of the metabolic pathways (oxidation and conjugation) are significantly reduced in neonates, with enzyme activity significantly lower in preterm neonates than in term neonates [14,15].

- Drug doses in relation to body weight need to be lower in neonates than in infants and children.
- Mechanisms of drug metabolism should be assessed in all age groups in which a drug is likely to be used in practice, and doses altered depending on findings.

## 5. Excipient Toxicity

All medicines contain excipients. Excipients are chemical substances that increase the solubility, stability or palatability of medicines. One of the earliest examples of excipient toxicity was the use of diethylene glycol as a solvent to make sulphonamides more soluble in water [16,17]. Diethylene glycol is a highly toxic substance and was responsible for the death of many children and adults in the USA in the 1930s [16].

Benzyl alcohol is an excipient that is used for its antibacterial properties and is a constituent of ampoules of sodium chloride and water that are administered intravenously. Unfortunately, the solutions contained 0.9% benzyl alcohol which proved to be toxic in premature neonates. The neonates were receiving multiple injections of benzyl alcohol-containing solutions through catheter flushes and reconstitution or dilution of medications [18]. The toxicity occurred in neonates because they have a reduced capacity to metabolise alcohol. Additionally, the amount of alcohol administered in relation to their body weight will have been far greater than that administered to a 70 kg adult. The exact number of deaths associated with the use of benzyl alcohol in ampoules of sodium chloride and water is unknown.

An intravenous vitamin E preparation (E-Ferol) was used to prevent retinopathy of prematurity in preterm neonates in several centres in the USA in 1983 [19]. Thirty-eight deaths were reported and the intravenous vitamin E preparation was withdrawn. It was suggested that excipients used were responsible for the toxicity [20]. These are examples from the last century, but a recent European study of potentially harmful excipients in neonatal medicines has shown it may be an ongoing problem [21]. The increasing research into the effect of excipients is to be welcomed and can only improve the safety of the use of medicines in children [21].

- All medicines contain excipients, which are added to the active product and may be toxic. When adverse events occur then excipients should be considered as well as the constituent drug.

## 6. Drug Interactions

There have been several case reports of calcium precipitation in the lungs of neonates and young infants in association with the use of ceftriaxone and calcium containing solutions [22,23]. The exact mechanism of this interaction is uncertain. Enzyme inhibition by one drug may result in an enhanced effect of another drug, e.g., erythromycin inhibiting CYP3A4 metabolism of midazolam, resulting in enhanced sedation [24]. Drug interactions are a potential problem in children receiving long term medication, especially antiepileptic drugs [25]. An example of a drug interaction involving anti-epileptic drugs is the use of lamotrigine in conjunction with valproic acid. The main ADR associated with lamotrigine is a skin reaction [26]. In the majority of cases the skin reactions are mild. There is a risk however of Stevens-Johnson syndrome or toxic epidermal necrolysis. The use of valproic acid in conjunction with lamotrigine significantly increases the risk of skin reactions [26]. The mechanism for this interaction is also unknown. An awareness that there is always a risk of a drug interaction, when prescribing more than one medicine, should always be present in the prescribers mind.

- Check for possible drug interactions in a formulary before prescribing multiple medications.
- Avoid using lamotrigine in combination with valproic acid whenever possible.

## 7. Polypharmacy

Polypharmacy significantly increases the risk of drug toxicity [26,27,28] and therefore should be avoided wherever possible. The concept of using more than one medicine to treat a condition in order to minimise drug toxicity was initially developed for the management of pain relief [29]. This use of polypharmacy was designed to reduce the dosage of opioid analgesics which are known to be associated with significant toxicity. The use of polypharmacy in relation to analgesia has been shown to be effective both in terms of insuring adequate pain relief and also in relation to reducing toxicity [29]. Unfortunately, polypharmacy has also been introduced in other therapeutic areas where it increases the risk of toxicity.

Valproic acid is a highly effective anticonvulsant and is the drug of choice for many children with epilepsy. Hepatotoxicity is a rare ADR associated with sodium valproate. Case reports of hepatoxicity in association with valproic acid were reported in the late 1970s. A review of 37 cases of fatal hepatoxicity in the USA identified three major risk factors: polytherapy, developmental delay and an age of under three years [30] A review of individual case safety reports to the WHO collaborating centre for international drug monitoring in Uppsala identified hepatotoxicity as the most likely cause of death in association with valproic acid in children [27]. Again polytherapy was a significant risk factor and the risk of hepatotoxicity appeared to be greatest in children aged six years and below, but can occur at any age [27].

- Polytherapy with valproic acid increases the risk of hepatotoxicity.

## 8. Unknown Mechanisms

There are numerous cases of drug toxicity where the mechanism is unknown. In these situations drug toxicity can be minimised by avoiding the use of certain medications for certain conditions.

### 8.1. Salicylates and Reye’s Syndrome

Reye’s syndrome is a life-threatening illness with drowsiness, seizures, hypoglycaemia and liver failure. It was first described in 1963 and a possible link between Reye’s syndrome and salicylates was postulated in 1965 [31]. Giles noted that 15 of 31 cases of Reye’s syndrome had received salicylates prior to admission [31]. The association between Reye’s syndrome and the use of salicylates as an antipyretic during a viral infection was confirmed in 1980 [32]. Subsequently salicylates were contraindicated as an antipyretic and analgesic in children ≤12 years in many countries. This has led to a large reduction in the incidence of Reye’s syndrome [33]. Subsequently it has been reported in children between the ages of 12 and 16 and so is no longer recommended in this age group as an antipyretic [34]. Although the mechanism of action is unknown, by avoiding the use of salicylates in children with a viral illness one has been able to dramatically reduce the incidence of Reye’s syndrome.

- Avoid salicylates as an antipyretic in children of all ages.

### 8.2. Propofol and Metabolic Acidosis

Propofol is a short acting anaesthetic agent that is safe and effective as an anaesthetic agent. It was used as an intravenous sedative in critically ill children due to its short acting properties. Children receiving propofol as a sedative agent received considerably higher cumulative doses of propofol than when used as an anaesthetic agent. Unfortunately, several children died following the use of propofol as a sedative agent [35]. The children developed severe metabolic acidosis and lipaemia. Although the mechanism responsible for the metabolic acidosis remains uncertain, toxicity is more likely with higher doses [36].

- Avoid the use of propofol as a sedative in critically ill children.

### 8.3. Vigabatrin and Visual Field Defects

Vigabatrin is an antiepileptic drug that is particularly effective in the management of infantile spasms. Shortly after its initial use, however, constrictions of the visual fields were described in both adults and children [37]. The risk of visual field defects is thought to be lower in children than in adults. Corticosteroids are now the first line treatment for children with infantile spasms but vigabatrin may be necessary in children who do not respond to corticosteroids. Monitoring for visual field defects is important in these children.

- Vigabatrin use in children with infantile spasms may be justified but monitoring for visual field defects is essential.

### 8.4. Corticosteroids and Growth

Corticosteroids are used in a wide variety of medical conditions. They have been shown to be effective in the management of asthma. A particular problem associated with their use in children is their adverse effect on growth. The majority of children receiving corticosteroids for the management of their asthma receive inhaled corticosteroids in order to minimise toxicity. Growth suppression is, however, still a potential problem despite the administration of corticosteroids by the inhaled route and children with symptoms and those taking high doses should be screened for adrenal suppression [38].

- Be aware of the potential adverse effect on growth of inhaled corticosteroids.

### 8.5. Corticosteroids and the Developing Brain

The corticosteroid dexamethasone has been used in preterm neonates with hyaline membrane disease in order to reduce the risk of chronic lung disease. Although effective in reducing the risk of chronic lung disease, post-natal dexamethasone has resulted in an increase in adverse neurological outcomes [39,40,41]. The mechanism of neurotoxicity is uncertain. The neurotoxicity however does highlight the increased susceptibility of preterm neonates to drug toxicity that is not found in other age groups.

- Be aware that preterm neonates may experience different adverse drug reactions to other age groups.

### 8.6. Macrolides and Pyloric Stenosis

Erythromycin is an old antibiotic that has been used extensively. Its association with pyloric stenosis was first reported in the 1970s [42]. Subsequently, others confirmed the association [43]. It has since been recognised as an uncommon adverse drug reaction associated with erythromycin. Azithromycin is a newer macrolide antibiotic that is widely used. Its advantages over erythromycin are that it can be administered once daily and is less likely to cause vomiting. It too, however, appears to be associated with the development of pyloric stenosis [44,45].

- Be aware that some adverse drug reactions may be a risk with the class of drug used rather than just the individual drug.

## 9. Rational Use of Medicines

Medicines are essential for the management of many diseases and illnesses effecting children. It is important, however, to recognise that many medicines are used inappropriately [46]. Antibiotics are the most extensively used drug in children. Two recent studies in France and Serbia reported that in both countries, antibiotics are over-prescribed [47,48]. Over 85% of children in Serbia with a viral upper respiratory tract infection received an antibiotic [48]. Over half the children in France received an antibiotic each year. Other drugs were also used irrationally. In France, one in five children under the age of two years received domperidone [47]. The inappropriate use of domperidone and protein pump inhibitors for infants with mild gastro-oesophageal reflux has been highlighted recently [49].

The rational use of medicines in children has been a neglected issue in high income countries as well as countries with limited resources [50]. The exclusive use of cough and cold medication in infants in the United States of America was associated with significant toxicity including fatalities [51]. It is important to ensure that there is an evidence base for the use of every single medicine that is prescribed to children.

- Avoid prescribing unnecessary medicines.

## 10. Conclusions

Drug toxicity should always be considered as a cause of a child’s symptoms and the risk of possible harm should be considered before prescribing a medicine. Discussion should always take place with children and their parents about the risk of the medicines they are being prescribed and their common adverse affects. By using medicines in a rational manner, drug toxicity can hopefully be minimised.
